# A 2 year physical activity and dietary intervention attenuates the increase in insulin resistance in a general population of children: the PANIC study

**DOI:** 10.1007/s00125-020-05250-0

**Published:** 2020-08-20

**Authors:** Timo A. Lakka, Niina Lintu, Juuso Väistö, Anna Viitasalo, Taisa Sallinen, Eero A. Haapala, Tuomo T. Tompuri, Sonja Soininen, Panu Karjalainen, Theresia M. Schnurr, Santtu Mikkonen, Mustafa Atalay, Tuomas O. Kilpeläinen, Tomi Laitinen, David E. Laaksonen, Kai Savonen, Soren Brage, Ursula Schwab, Jarmo Jääskeläinen, Virpi Lindi, Aino-Maija Eloranta

**Affiliations:** 1 Institute of Biomedicine, School of Medicine, University of Eastern Finland, Kuopio Campus, P.O. Box 1627, FI-70211 Kuopio, Finland; 2 Department of Clinical Physiology and Nuclear Medicine, Kuopio University Hospital, University of Eastern Finland, Kuopio, Finland; 3 Foundation for Research in Health Exercise and Nutrition, Kuopio Research Institute of Exercise Medicine, Kuopio, Finland; 4 Institute of Dentistry, University of Eastern Finland, Kuopio, Finland; 5 Institute of Public Health and Clinical Nutrition, University of Eastern Finland, Kuopio, Finland; 6 Faculty of Sport and Health Sciences, University of Jyväskylä, Jyväskylä, Finland; 7 Social and Health Center, City of Varkaus, Finland; 8 Novo Nordisk Foundation Center for Basic Metabolic Research, Faculty of Health and Medical Sciences, University of Copenhagen, Copenhagen, Denmark; 9 Department of Applied Physics, University of Eastern Finland, Kuopio, Finland; 10 Department of Medicine, Endocrinology and Clinical Nutrition, Kuopio University Hospital, Kuopio, Finland; 11 MRC Epidemiology Unit, University of Cambridge, Cambridge, UK; 12 Department of Pediatrics, Institute of Clinical Medicine, Kuopio University Hospital and University of Eastern Finland, Kuopio, Finland; 13 University of Eastern Finland Library Kuopio, Kuopio, Finland

**Keywords:** Body fat, Children, Diet, Glucose, HOMA-IR, Insulin, Intervention, Lean body mass, Physical activity, Sedentary time

## Abstract

**Aims/hypothesis:**

We studied for the first time the long-term effects of a combined physical activity and dietary intervention on insulin resistance and fasting plasma glucose in a general population of predominantly normal-weight children.

**Methods:**

We carried out a 2 year non-randomised controlled trial in a population sample of 504 children aged 6–9 years at baseline. The children were allocated to a combined physical activity and dietary intervention group (306 children at baseline, 261 children at 2-year follow-up) or a control group (198 children, 177 children) without blinding. We measured fasting insulin and fasting glucose, calculated HOMA-IR, assessed physical activity and sedentary time by combined heart rate and body movement monitoring, assessed dietary factors by a 4 day food record, used the Finnish Children Healthy Eating Index (FCHEI) as a measure of overall diet quality, and measured body fat percentage (BF%) and lean body mass by dual-energy x-ray absorptiometry. The intervention effects on insulin, glucose and HOMA-IR were analysed using the intention-to-treat principle and linear mixed-effects models after adjustment for sex, age at baseline, and pubertal status at baseline and 2 year follow-up. The measures of physical activity, sedentary time, diet and body composition at baseline and 2 year follow-up were entered one-by-one as covariates into the models to study whether changes in these variables might partly explain the observed intervention effects.

**Results:**

Compared with the control group, fasting insulin increased 4.65 pmol/l less (absolute change +8.96 vs +13.61 pmol/l) and HOMA-IR increased 0.18 units less (+0.31 vs +0.49 units) over 2 years in the combined physical activity and dietary intervention group. The intervention effects on fasting insulin (regression coefficient β for intervention effect −0.33 [95% CI −0.62, −0.04], *p* = 0.026) and HOMA-IR (β for intervention effect −0.084 [95% CI −0.156, −0.012], *p* = 0.023) were statistically significant after adjustment for sex, age at baseline, and pubertal status at baseline and 2 year follow-up. The intervention had no effect on fasting glucose, BF% or lean body mass. Changes in total physical activity energy expenditure, light physical activity, moderate-to-vigorous physical activity, total sedentary time, the reported consumption of high-fat (≥60%) vegetable oil-based spreads, and FCHEI, but not a change in BF% or lean body mass, partly explained the intervention effects on fasting insulin and HOMA-IR.

**Conclusions/interpretation:**

The combined physical activity and dietary intervention attenuated the increase in insulin resistance over 2 years in a general population of predominantly normal-weight children. This beneficial effect was partly mediated by changes in physical activity, sedentary time and diet but not changes in body composition.

**Trial registration:**

ClinicalTrials.gov NCT01803776

**Figure:**
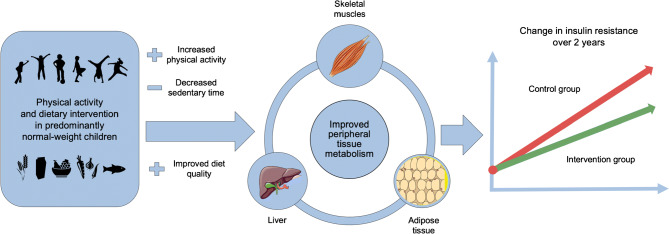
Graphical abstract

**Electronic supplementary material:**

The online version of this article (10.1007/s00125-020-05250-0) contains supplementary material, which is available to authorized users.

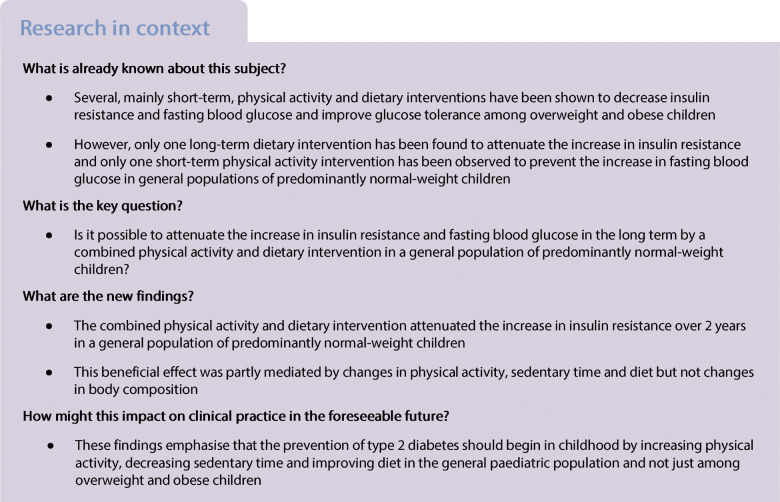



## Introduction

The number of people with type 2 diabetes and prediabetes has increased dramatically worldwide over the past decades [1]. Their prevalence has increased in many countries among children and adolescents [2]. This alarming trend is likely due to decreased physical activity, increased sedentary time, unhealthy diet and consequently increased body fat content [1–3]. Insulin resistance, which is crucial in the pathogenesis of type 2 diabetes, is usually evident long before hyperglycaemia, is strongly associated with adiposity, and strongly predicts future risk of type 2 diabetes and cardiovascular disease [4]. Pathophysiological processes underlying type 2 diabetes, such as insulin resistance, may begin during the fetal period [2]. There is a marked increase in insulin resistance during pubertal development [5] but it has been found to rise progressively already some years before puberty [6]. All this evidence emphasises the need for the prevention of type 2 diabetes since childhood.

Several physical activity [7–12] and dietary [13] interventions, particularly in combinations [13–19] and mainly short-term, have been shown to decrease insulin resistance among overweight and obese children. Moreover, a few combined physical activity and dietary interventions have been found to decrease fasting blood glucose [13] and improve glucose tolerance [15–17] in overweight and obese children. Some of these lifestyle interventions have also been observed to decrease adiposity [13, 15, 16], suggesting that their beneficial effects on insulin resistance, fasting blood glucose and glucose tolerance in overweight and obese children are at least partly mediated by reductions in body fat percentage (BF%). However, the results of some studies suggest that physical activity interventions decrease insulin resistance among children even without a change in BF% or lean body mass [9, 12].

Evidence on the long-term effects of physical activity and dietary interventions on insulin resistance and blood glucose in general populations of children, most of whom have a normal body weight, is needed to provide insight into the role of lifestyle changes in the early prevention of type 2 diabetes. It is also important to determine whether changes in physical activity, sedentary time, diet and body composition might mediate the beneficial effects of the long-term lifestyle interventions on insulin resistance and blood glucose to plan effective strategies for the early prevention of type 2 diabetes. To our knowledge, only one long-term dietary intervention has been found to attenuate the increase in insulin resistance [20] and only one short-term physical activity intervention has been observed to prevent the increase in fasting blood glucose [21] in general populations of predominantly normal-weight children.

We therefore carried out a 2 year controlled trial to investigate the long-term effects of a combined physical activity and dietary intervention on fasting serum insulin, fasting plasma glucose and HOMA-IR in a general population of children, most of whom had a normal body weight. We also examined whether 2 year changes in physical activity, sedentary time, diet, BF% and lean body mass might partly mediate the observed intervention effects.

## Methods

### Study design and participants

The Physical Activity and Nutrition in Children (PANIC) study is a non-randomised controlled trial on the effects of a combined physical activity and dietary intervention on cardiometabolic risk factors and other health outcomes in a population sample of children from the city of Kuopio, Finland [22, 23]. The Research Ethics Committee of the Hospital District of Northern Savo approved the study protocol in 2006 (Statement 69/2006). The parents or caregivers of the children gave their written informed consent, and the children provided their assent to participation. The PANIC study has been carried out in accordance with the principles of the Declaration of Helsinki as revised in 2008.

**Fig. 1:**
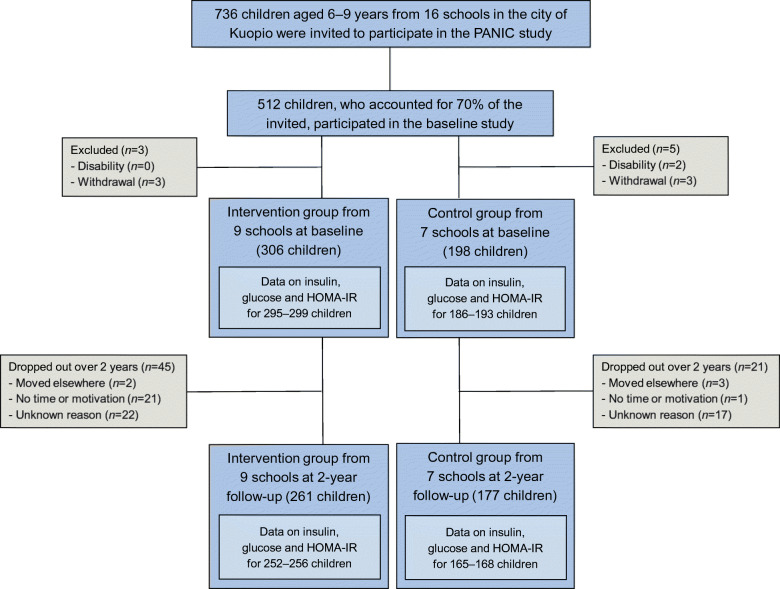
Flowchart of the PANIC study

We invited 736 children aged 6–9 years who started the first grade in 16 primary schools of the city of Kuopio in 2007–2009 to participate in the study (Fig. 1). Altogether, 512 (70%) children (248 girls, 264 boys) accepted the invitation and participated in the baseline examinations between October 2007 and December 2009. The participants did not differ in sex, age, height-SD score (SDS) or BMI-SDS from all children who started the first grade in the city of Kuopio in 2007–2009. We excluded six children from the study at baseline either owing to their physical disabilities that could hamper participation in the intervention or withdrawal of the families because they had no time or motivation to attend the study. We also excluded data from two children whose parents or caregivers later withdrew their permission to use these data in the study. The final study sample thus included 504 children at baseline.

We allocated the children from nine schools to a combined physical activity and dietary intervention group (306 children, 60%) and the children from seven schools to a control group (198 children, 40%) to avoid contamination in the control group by any local or national health promotion programmes that could have been initiated in the study region during the follow-up period. We also proportionally matched the intervention and control group according to the location of the schools (urban vs rural) to minimise sociodemographic differences between the groups. We included more children in the intervention group than in the control group because of a larger number of dropouts expected in the intervention group and to retain a sufficient statistical power for comparison between the groups. The children, their parents or caregivers, or people carrying out the examination visits or doing the measurements were not blinded to the group assignment. A total of 261 children (85% of those invited) from the intervention group and 177 (89%) children from the control group participated in the 2 year follow-up examinations between November 2009 and January 2012. The median (interquartile range) of follow-up time was 2.1 (2.1–2.2) years in the intervention and control group.

Data on fasting serum insulin and HOMA-IR were available in the intervention group for 295 children at baseline and 252 children at 2 year follow-up and in the control group for 186 children at baseline and 165 children at 2 year follow-up. Data on fasting plasma glucose were available in the intervention group for 299 children at baseline and 256 children at 2 year follow-up and in the control group for 193 children at baseline and 168 children at 2 year follow-up. The partly incomplete data on these outcome variables were due either to missing fasting blood samples for the insulin and glucose analyses or to haemolysis that interfered with the insulin analyses.

### Physical activity and dietary intervention

The 2 year individualised and family-based physical activity and dietary intervention consisted of six intervention visits that occurred 0.5, 1.5, 3, 6, 12 and 18 months after baseline examinations. Each intervention visit included 30–45 min of physical activity counselling and 30–45 min of dietary counselling for the children and their parents or caregivers. The children and their parents or caregivers received individualised advice from a specialist in exercise medicine and a clinical nutritionist on how to increase physical activity, decrease sedentary time and improve the diet of the children in everyday conditions. Each visit had a specific topic of discussion (physical activity, sedentary time and diet; electronic supplementary material [ESM] Table 1) in accordance with the goals of the intervention (ESM Table 2) and included practical tasks on these topics for the children. The children and their parents or caregivers also received fact sheets on physical activity, sedentary time and diet, and verbal and written information on opportunities for exercising in the city of Kuopio. Some material support was also given for physical activity, such as exercise equipment and allowance for playing indoor sports. Of the 306 children in the intervention group who attended the baseline examination, 266 (87%) participated in all six visits, 281 (92%) in at least five visits, and 295 (96%) in at least four visits. The children in the intervention group, particularly those who did not attend organised sports or exercise, were also encouraged to participate in after-school exercise clubs organised at the nine schools by trained exercise instructors of the PANIC study. The children in the control group were not allowed to attend these exercise clubs to avoid a non-intentional intervention in the control group. Altogether, 254 (83%) of the 306 children in the intervention group participated in at least one of the after-school exercise clubs, and 124 (41%) participated at least once a month. In the control group, the children and their parents or caregivers received general verbal and written advice on health-improving physical activity and diet only at baseline with no further lifestyle counselling.

### Measurement of insulin, glucose and HOMA-IR

A research nurse took blood samples in the morning, after children had fasted overnight for at least 12 h. Serum insulin was analysed using an electrochemiluminescence immunoassay with the sandwich principle (Roche Diagnostics, Mannheim, Germany). The within-day and between-day coefficients of variation for the insulin analyses were 1.3–3.5% (76–1104 pmol/l) and 1.6–4.4% (132–681 pmol/l), respectively. A hexokinase method was used to analyse plasma glucose (Roche Diagnostics). The within-day and between-day coefficients of variation for the glucose analyses were 0.7–0.9% (5.1–11.9 mmol/l) and 1.5–1.8% (3.4–14.1 mmol/l), respectively. HOMA-IR was calculated as explained elsewhere [24].

### Assessment of physical activity and sedentary time

The measures of physical activity and sedentary time, reflecting the goals of the intervention, including average daily total physical activity energy expenditure, light, moderate and vigorous physical activity and total sedentary time, were assessed using individually calibrated combined heart rate and body movement monitoring [25]. The methods used for the assessment of physical activity and sedentary time [26–29] are explained in detail in ESM Methods. Average total physical activity energy expenditure was calculated in kJ/kg daily. Light, moderate and vigorous physical activity were defined as time spent at intensity >1.5 and ≤4.0 metabolic equivalents (METs), >4.0 and ≤7.0 METs and >7.0 METs, respectively, where one MET is defined as an energy expenditure of 71 J kg^−1^ min^−1^ or oxygen uptake of 3.5 ml kg^−1^ min^−1^. Moderate-to-vigorous physical activity was calculated by summing moderate and vigorous physical activity. Total sedentary time was defined as the time spent at intensity ≤1.5 METs, excluding sleep.

### Assessment of dietary factors

Dietary factors reflecting the goals of the intervention, including the consumption of vegetables, fruit and berries, high-fibre (≥5%) grain products, low-fibre (<5%) grain products, high-fat (≥60%) vegetable oil-based spreads, vegetable oils, butter-based spreads, high-fat (≥1%) milk, low-fat (<1%) milk, red meat, fish and foods with high sugar content, were assessed using 4 day food records [30]. (See ESM Methods for details.) We used the Finnish Children Healthy Eating Index (FCHEI) as an indicator of overall diet quality [31]. The index was calculated by summing the reported consumption of the following foods based on their quantiles in the present study population [30]: vegetables, fruit and berries (scored 1–10); high-fat (≥60%) vegetable oil-based spreads and vegetable oils (0–10); low-fat (<1%) milk (0–9); fish (0–6); and foods with high sugar content (10–1). The index thus ranged between 2 and 45, a higher score indicating higher overall diet quality.

### Assessment of body size and composition

Body height and weight were assessed, BMI was calculated, age- and sex-standardised height-SDS and BMI-SDS were calculated, overweight and obesity were defined, and BF% and lean body mass were measured [25, 30]. (See ESM Methods for details.)

### Assessment of pubertal status

A research physician assessed pubertal status according to breast development for girls (scored M 1–5) and according to testicular volume measured by an orchidometer for boys (scored G 1–5) using the Tanner staging method [32, 33].

### Statistical methods

We performed all statistical analyses using the IBM SPSS Statistics software, version 25.0 (IBM Corp., Armonk, NY, USA). A *p* value of <0.05 for a two-tailed test was used to indicate statistical significance. The outcome variables were normally distributed based on visual observation of the histograms. We compared baseline characteristics between the intervention and control group by linear mixed-effects models with cluster-robust SEs, except body weight status for which comparison was performed by generalised linear mixed-effects models with ordered structure, to account for the clustering effect of schools. Sample size calculations [22, 23] are explained in ESM Methods. We studied the effects of the combined physical activity and dietary intervention on insulin, glucose and HOMA-IR using the intention-to-treat principle by including all 504 children in the statistical analyses. We analysed the data using linear mixed-effects models according to a three-level data structure by clustering the repeated outcome variables at baseline and 2 year follow-up within children who were considered as being included in the mixed model structure and were clustered within schools. However, we did not use the three-level data structure in the final models because allowing for school-level clustering did not improve model fit based on the Bayesian information criterion, as presented in ESM Methods. We adjusted the data for sex, age at baseline, and pubertal status at baseline and 2 year follow-up and included main effects for time and for study group × time interaction in the models. The mixed-effects models assume that the data are missing at random. We found that this assumption was reasonable because there were only minor differences in the characteristics between children with the outcome data and children without these data at 2 year follow-up. We did not include study group as a separate variable in the models because there were no statistically significant differences in insulin, glucose and HOMA-IR between the intervention and control group at baseline. The formula for the linear mixed-effects model is presented in ESM Methods. We also examined whether 2 year changes in physical activity, sedentary time, diet, BF% and lean body mass might partly explain the observed intervention effects on insulin, glucose and HOMA-IR by calculating percentage changes in regression coefficients for differences in estimated changes in insulin, glucose and HOMA-IR between the intervention and control group after adding physical activity, sedentary time, diet, BF% and lean body mass at baseline and 2 year follow-up one-by-one as covariates into the linear mixed-effects models.

## Results

### Characteristics of children

There were no differences in baseline characteristics between the groups, except that children in the combined physical activity and dietary intervention group consumed less high-fat milk than children in the control group (Table 1). Only 2–3% of the children at baseline (Table 1) and 23–24% of the children at 2 year follow-up (ESM Table 3) had entered puberty (Tanner stage ≥2). Altogether, 13% of the children in both groups were overweight or obese at baseline (Table 1), whereas 17% of the children in the intervention group and 18% of the children in the control group were overweight or obese at 2 year follow-up (ESM Table 3).

**Table 1:** Baseline characteristics of children in the physical activity and dietary intervention and control groups

Characteristic	Intervention group (*n* = 306)	Control group (*n* = 198)	*p* value
Sex			0.520
Boys, *n* (%)	162 (52.9)	99 (50.0)	
Girls, *n* (%)	144 (47.1)	99 (50.0)	
Age, years	7.6 ± 0.4	7.6 ± 0.4	0.989
Pubertal status, *n* (%)			0.588
Tanner stage 1	298 (97.4)	194 (98.0)	
Tanner stage 2	8 (2.6)	4 (2.0)	
Body weight, kg	27.0 ± 4.8	26.8 ± 5.3	0.783
Body height, cm	128.9 ± 5.5	128.6 ± 5.9	0.847
Body height-SDS	0.15 ± 0.99	0.12 ± 1.04	0.713
BMI-SDS	−0.16 ± 1.06	−0.20 ± 1.11	0.658
Body weight status, *n* (%)			0.751
Normal weight	264 (86.3)	173 (87.4)	
Overweight	30 (9.8)	15 (7.6)	
Obesity	12 (3.9)	10 (5.1)	
BF%	19.8 ± 8.3	19.9 ± 8.2	0.893
Lean body mass, kg	20.7 ± 2.4	20.5 ± 2.5	0.784
Total PA energy expenditure, kJ kg^−1^ day^−1^	101 ± 32	95 ± 34	0.514
Light PA, h/day	8.6 ± 1.8	8.3 ± 1.8	0.216
Moderate-to-vigorous PA, h/day	2.0 ± 1.0	1.8 ± 1.1	0.648
Sedentary time, h/day	3.8 ± 2.0	4.1 ± 2.3	0.362
FCHEI	23.6 ± 6.9	22.6 ± 7.0	0.269
Food consumption, g/day			
Vegetables, fruit and berries	203 ± 114	219 ± 119	0.159
High-fibre (≥5%) grain products^a^	63 ± 39	62 ± 40	0.909
Low-fibre (<5%) grain products^b^	113 ± 54	115 ± 51	0.644
High-fat (60–80%) vegetable oil-based spreads	6.9 ± 7.6	7.7 ± 8.6	0.362
Vegetable oils	4.3 ± 4.4	3.8 ± 3.8	0.271
Butter-based spreads	5.8 ± 7.2	6.1 ± 7.2	0.665
High-fat (≥1%) milk	170 ± 211	222 ± 243	0.019
Low-fat (<1%) milk	370 ± 289	393 ± 299	0.441
Red meat	56 ± 29	58 ± 34	0.442
Fish	15 ± 20	16 ± 23	0.807
Foods with high sugar content^c^	184 ± 135	207 ± 147	0.275
Total energy intake, MJ/day	6.8 ± 1.3	7.0 ± 1.3	0.254
Percentage of daily energy intake			
Carbohydrates	52.0 (4.8)	51.5 (5.4)	0.847
Sucrose	12.7 (3.6)	12.7 (3.7)	0.932
Total fat	29.7 (4.8)	30.4 (5.3)	0.516
Saturated fat	12.0 (2.7)	12.4 (2.9)	0.520
Monounsaturated fat	9.9 (1.7)	10.1 (2.0)	0.588
Polyunsaturated fat	4.9 (1.2)	4.9 (1.3)	0.734
Protein	16.9 (2.4)	16.7 (2.5)	0.481
Fibre intake, g/day	14.5 ± 4.1	14.4 ± 4.0	0.932

The values are unadjusted means ± SD for continuous variables and *n* (%) for categorical variables

Baseline data on sex, age, pubertal status, body weight, body height, body height-SDS, BMI-SDS and body weight status were available for all 306 children in the intervention group and for all 198 children in the control group. Data on variables were available for the following numbers of children in the intervention group and control group, respectively: BF% and lean body mass 298 and 195; total physical activity energy expenditure 290 and 188; light and moderate-to-vigorous physical activity 276 and 175; sedentary time 274 and 175 children; and dietary factors 256 and 167. The food consumption and total energy intake, macronutrient intake and dietary fibre intake were calculated from reported food consumption from 4 day food records filled out by the parents or caregivers of the children

*p* values are shown for differences between the intervention and control group from linear mixed-effects models with cluster-robust SEs, except that numbers (percentages) for body weight status and *p* values for their differences between the intervention and control group are from generalised linear mixed-effects models with ordered structure, to account for the clustering effect of schools. Differences with *p* values <0.05 were considered statistically significant

^a^Wholegrain pasta, rice and oatmeal

^b^White pasta, rice and flour

^c^Sugar-sweetened beverages, fruit juice, candies, chocolate, added sugar, ice cream, puddings, pastries and biscuits

PA, physical activity

### Intervention effects on insulin, glucose, HOMA-IR, BF% and lean body mass

Fasting insulin increased 4.65 pmol/l less (absolute change +8.96 vs +13.61 pmol/l) and HOMA-IR increased 0.18 units less (+0.31 vs +0.49 units) over 2 years in the combined physical activity and dietary intervention group than in the control group (Table 2). These intervention effects on fasting insulin and HOMA-IR were statistically significant after adjustment for sex, age at baseline, and pubertal status at baseline and 2 year follow-up (Table 2). However, the intervention had no effect on fasting glucose (Table 2), BF% (regression coefficient β for intervention effect −0.02 [95% CI −0.39, 0.36], *p* = 0.926) or lean body mass (β for intervention effect 0.03 [95% CI −0.07, 0.13], *p* = 0.567) over 2 years.

**Table 2:** Effects of combined physical activity and dietary intervention on fasting insulin, fasting glucose and HOMA-IR over 2 years

Variable	Intervention group		Control group		Difference in estimated change between groups	
	Baseline	2 year follow-up	Baseline	2 year follow-up	β (95% CI)	*p* value
Fasting insulin, pmol/l	30.70 (16.25)	39.66 (21.46)	31.95 (16.74)	45.56 (26.95)	−0.33 (−0.62, −0.04)	0.026
Fasting glucose, mmol/l	4.81 (0.37)	5.00 (0.50)	4.84 (0.43)	5.05 (0.49)	−0.03 (−0.07, 0.02)	0.263
HOMA-IR	0.97 (0.55)	1.28 (0.74)	1.00 (0.57)	1.49 (0.97)	−0.084 (−0.156, −0.012)	0.023

The measured values are presented as means±SD; regression coefficient β (95% CI) values are shown for differences in estimated changes in insulin, glucose and HOMA-IR between the intervention and control group and *p* values for intervention effects are received from linear mixed-effects models adjusted for sex, age at baseline, and pubertal status at baseline and 2 year follow-up

Data on insulin and HOMA-IR were available in the intervention group for 295 children at baseline and 252 children at 2 year follow-up and in the control group for 186 children at baseline and 165 children at 2-year follow-up. Data on glucose were available in the intervention group for 299 children at baseline and 256 children at 2 year follow-up and in the control group for 193 children at baseline and 168 children at 2 year follow-up. The partly incomplete data on these outcome variables were either due to missing fasting blood samples for the insulin and glucose analyses or to haemolysis that interfered with the insulin analyses

### Mediators for intervention effects on insulin and HOMA-IR

The intervention effects over 2 years on fasting insulin and HOMA-IR, respectively, were explained by the following changes (β for intervention effect was changed by the % shown): total physical activity energy expenditure and moderate-to-vigorous physical activity 39% and 38%; light physical activity 33% and 33%; total sedentary time 30% and 31%; the reported consumption of high-fat (≥60%) vegetable oil-based spreads 21% and 23%; and FCHEI 12% and 11% (Table 3). However, 2 year changes in other dietary factors, BF% or lean body mass did not explain any of the intervention effects (Table 3).

**Table 3:** Changes in physical activity, sedentary time, diet, BF% and lean body mass as mediators for the beneficial effects of combined physical activity and dietary intervention on fasting insulin and HOMA-IR over 2 years

Adjustment	Fasting insulin		HOMA-IR	
	β for intervention effect	Percentage of β for intervention effect explained	β for intervention effect	Percentage of β for intervention effect explained
Sex, age at baseline, and pubertal status at baseline and 2 year follow-up	−0.33		−0.084	
+ Total PA energy expenditure, kJ kg^−1^ day^−1^	−0.20	39	−0.052	38
+ Light PA, h/day	−0.22	33	−0.056	33
+ Moderate-to-vigorous PA, h/day	−0.20	39	−0.052	38
+ Sedentary time, h/day	−0.23	30	−0.058	31
+ FCHEI	−0.29	12	−0.075	11
+ High-fat (≥60%) vegetable oil-based spreads, g/day	−0.26	21	−0.065	23
+ BF%	−0.33	0	−0.084	0
+ Lean body mass, kg	−0.34	−3	−0.086	−2

The values are regression coefficients β for differences in estimated changes in insulin and HOMA-IR between the intervention and control group from linear mixed-effects models adjusted for sex, age at baseline, and pubertal status at baseline and 2 year follow-up and percentage changes in regression coefficients β after additional adjustment for the measures of physical activity, sedentary time, diet quality and body composition, indicating whether these variables partly explained the beneficial effects of the combined physical activity and dietary intervention on fasting serum insulin and HOMA-IR. Data on variables were available for the following numbers of children at baseline and 2 year follow-up, respectively: insulin and HOMA-IR 295 and 252 in the intervention group, and 186 and 165 in the control group; sex, age and pubertal status 306 and 261 children in the intervention group, and 198 and 177 in the control group; total physical activity energy expenditure 290 and 224 in the intervention group, and 188 and 159 in the control group; light and moderate-to-vigorous physical activity 276 and 218 in the intervention group, and 175 and 156 in the control group; sedentary time 274 and 216 in the intervention group, and 175 and 154 in the control group; FCHEI and high-fat (≥60%) vegetable oil-based spreads 256 and 231 in the intervention group, and 167 and 158 in the control group; BF% and lean body 298 and 248 in the intervention group, and 195 and 169 in the control group

β, regression coefficient; PA, physical activity

## Discussion

This controlled trial showed that the combined physical activity and dietary intervention attenuated the increase in insulin resistance, assessed by fasting serum insulin and HOMA-IR, but had no effect on fasting plasma glucose over 2 years in a general population of children. The intervention effects on insulin resistance were partly mediated by changes in physical activity, sedentary time and diet but not by changes in BF% or lean body mass.

Several mainly short-term physical activity and dietary interventions, particularly in combinations, have been shown to decrease insulin resistance among overweight and obese children [7–19]. To our knowledge, however, only one long-term dietary intervention has been observed to attenuate the increase in insulin resistance in general populations of predominantly normal-weight children [20]. One reason for this inconsistency is that overweight and obese children are usually highly insulin resistant [34, 35] and thus more likely to show the beneficial effect of lifestyle interventions on insulin resistance than normal-weight children. We found that the combined physical activity and dietary intervention attenuated the increase in fasting insulin by 4.65 pmol/l (34%) and the increase in HOMA-IR by 0.18 units (37%) over 2 years in a population sample of children in which 85% had a normal body weight at baseline. This observation is important from a public health perspective by suggesting that it is possible to attenuate the increase in insulin resistance by lifestyle interventions not only in overweight and obese children but also in general populations of predominantly normal-weight children.

Mainly short-term combined physical activity and dietary interventions have been found to decrease fasting glucose [13] and improve glucose tolerance [15–17] among overweight and obese children. However, we are aware of only one study in which a short-term physical activity intervention was observed to prevent the increase in fasting glucose in a general population of predominantly normal-weight children [22]. This is not surprising because fasting plasma glucose levels are usually maintained over decades within a narrow range by insulin and other hormones that regulate glucose production and utilisation, particularly in normal-weight individuals [36]. In the current study, the combined physical activity and dietary intervention had no effect on fasting plasma glucose in children, most of whom had a normal body weight. Moreover, almost all of the children had normal fasting plasma glucose levels at baseline making it unlikely that the physical activity and dietary intervention would show a beneficial effect on the glucose levels.

We have previously observed that the combined physical activity and dietary intervention increased total physical activity, unsupervised physical activity and organised sports and attenuated an increase in using computer and playing video games over 2 years in the present study population of children [23]. Moreover, we recently reported that an increase in moderate-to-vigorous physical activity and a decrease in total sedentary time were associated with a decrease in fasting insulin during the 2 year follow-up in these children [26]. In the present study, the beneficial effect of the combined physical activity and dietary intervention on insulin resistance was partly mediated by changes in total physical activity energy expenditure, light and moderate-to-vigorous physical activity and total sedentary time. These findings suggest that it is possible to attenuate the increase in insulin resistance in general populations of predominantly normal-weight children by increasing physical activity and decreasing sedentary time. Increased physical activity may have a beneficial effect on insulin resistance through the following mechanisms: increasing capillary density in skeletal muscles and improving the delivery of glucose, insulin and oxygen into the muscle cells [37]; increasing the number of type IIa fibres and improving oxidative metabolism in skeletal muscles [38]; increasing the expression of GLUT 4 in skeletal muscles and enhancing insulin- and muscle contraction-stimulated glucose uptake into the muscle cells [39]; and activating a number of signalling proteins and key enzymes of glucose metabolism, particularly glycogen synthase and hexokinase, in skeletal muscle cells [39, 40]. Physical activity may also increase the uptake and oxidation of NEFA [41], reduce the concentrations of deleterious lipid metabolites, such as diacylglycerols and ceramides [42], decrease inflammation [43], and reduce lipid peroxidation [44] in skeletal muscles.

Previously, we observed that the combined physical activity and dietary intervention increased the reported consumption of vegetables, high-fat vegetable oil-based spreads and low-fat milk and prevented the increase in the reported consumption of butter-based spreads over 2 years in the current study population of children [22]. In the present study, the beneficial effect of the intervention on insulin resistance was slightly mediated by changes in the reported consumption of high-fat (≥60%) vegetable oil-based spreads and the overall diet quality. Consistent with our findings, the beneficial effect of dietary intervention on insulin resistance among predominantly normal-weight children participating in the Special Turku Coronary Risk Factor Intervention Project for Children (STRIP) was partly mediated by the lower intake of saturated fat and the higher intake of polyunsaturated fat and carbohydrates in the dietary intervention group [20]. Taken together, the results of the STRIP and our study suggest that it is to some extent possible to attenuate the increase in insulin resistance among children, most of whom have a normal body weight, by replacing some of the saturated fat with unsaturated fat in the diet. This dietary change may exert a beneficial effect on insulin resistance by improving the composition of fatty acids, decreasing the accumulation of harmful lipid metabolites, such as diacylglycerols and ceramides, increasing GLUT 4 expression, activating signalling proteins, increasing lipid oxidation [45], and improving mitochondrial function [46] in skeletal muscles.

The beneficial effects of lifestyle interventions on insulin resistance among overweight and obese children appear to be at least partly due to decreased adiposity [13, 15, 16]. The underlying reason is that being overweight is one of the main causes of insulin resistance in children [34, 35]. However, there is some evidence that physical activity interventions are able to decrease insulin resistance independent of a change in adiposity among overweight and obese children [9, 12] and that dietary interventions can attenuate the increase in insulin resistance without a marked change in BF% in general populations of predominantly normal-weight children [20]. Consistent with these findings, we observed that the beneficial effect of the combined physical activity and dietary intervention on insulin resistance in a general population of children was not mediated by a change in BF%. Moreover, we showed that the intervention effect was also independent of a change in lean body mass. Therefore, further intervention studies are needed to reveal the biological mechanisms by which physical activity and dietary changes attenuate the increase in insulin resistance in children independent of changes in body composition.

Insulin resistance increases particularly during puberty [5], but increases have been noted some years before puberty, at the age of 7 years [6]. Increased insulin resistance during and before puberty is partly due to increased body fat content but also to elevated serum levels of IGF-1, which is primarily regulated by growth hormone [7]. There is some evidence that physical activity attenuates the natural peak of insulin resistance occurring at the age of 12–13 years independent of pubertal status and BF% [47]. Moreover, the dietary intervention initiated in infancy in the STRIP attenuated the increase in insulin resistance until childhood [20] and even late adolescence [48] independent of pubertal status. Consistent with these findings, we observed that the combined physical activity and dietary intervention attenuated the increase in insulin resistance independent of pubertal status, BF% and lean body mass. However, very few children at baseline and less than one-fourth of the children at the 2 year follow-up examination had entered puberty (all of them being at early puberty), with no difference between the intervention and control group. Thus, pubertal status was not a major confounder in our study.

A strength of our study is that we had for the first time the opportunity to investigate whether a long-term combined physical activity and dietary intervention has beneficial effects on insulin resistance and fasting plasma glucose in a general population of children, most of whom have a normal body weight. The thorough assessment of physical activity and sedentary time using individually calibrated heart rate and body movement monitoring, dietary factors using a 4 day food record, and BF% and lean body mass using dual-energy x-ray absorptiometry allowed us to examine whether these intervention effects were mediated by real changes in the targeted behaviours and body composition. Body size and composition in our study sample of children were similar to those of the large national reference population [49], as indicated by height-SDS and BMI-SDS, which made it possible to generalise the results to other children of the same age in Finland. We emphasised the individual needs of the families and parental involvement, both of which have been observed to improve adherence of families to lifestyle interventions [50]. Only 15% of the children in the intervention group dropped out during the 2 year follow-up, and almost 90% of the children and their parents or caregivers participated in all six physical activity and dietary counselling session, suggesting that the intervention was well accepted by the participants.

A limitation of the study is that we did not randomly allocate the participants to the intervention and control group but instead allocated the children from nine schools to the intervention group and the children from seven schools to the control group to avoid contamination in the control group by local or national health promotion programmes that could have been initiated in the study region during the follow-up period. This type of allocation of the children to the study groups also enabled us to organise after-school exercise clubs as part of the intervention at the nine school premises and thus avoid a non-intentional intervention in the control group. We also matched the intervention and control group according to the location of the schools so that children from urban and rural areas were included in both groups to minimise sociodemographic differences between the groups. There were only minor differences in baseline characteristics between the intervention and control group, suggesting fair success in avoiding selection bias. Moreover, we analysed the data using linear mixed-effects models that allowed us to control for the possible clustering effect of the schools.

This 2 year controlled trial showed that the combined physical activity and dietary intervention attenuated the increase in insulin resistance in a general population of predominantly normal-weight children and that this beneficial effect was partly mediated by changes in physical activity, sedentary time and diet. These findings emphasise that the prevention of type 2 diabetes should begin in childhood by increasing physical activity, decreasing sedentary time and improving diet in the general paediatric population and not just among overweight and obese children.

## Electronic supplementary material

ESM 1 (PDF 590 kb)

## Data Availability

Information about the PANIC study and the data used in the present paper is available at www.panicstudy.fi/en/etusivu. The data are not publicly available due to research ethical reasons and because the owner of the data is the University of Eastern Finland and not the research group. However, the corresponding author can provide further information on the PANIC study and the PANIC data on a reasonable request.

## Abbreviations

BF%: Body fat percentage
FCHEI: Finnish Children Healthy Eating Index
MET: Metabolic equivalent
PANIC: Physical Activity and Nutrition in Children
SDS: SD score
STRIP: Special Turku Coronary Risk Factor Intervention Project for Children
