# Supplementary material for: A 2 year physical activity and dietary intervention attenuates the increase in insulin resistance in a general population of children: the PANIC study
Source: Diabetologia. 2020 Aug 20;63(11):2270–81. doi: 10.1007/s00125-020-05250-0 (PMC7527318; doi:10.1007/s00125-020-05250-0)
Supplement: Supplementary file 1 — (PDF 590 kb) [file 125_2020_5250_MOESM1_ESM.pdf]

Electronic supplementary material (ESM)

Methods

Assessment of physical activity and sedentary time

The measures of physical activity and sedentary time reflecting the goals of the intervention, including average daily total physical activity energy expenditure, light, moderate and vigorous physical activity and total sedentary time, were assessed using a combined heart rate and body movement monitor called Actiheart® [1]. The monitor is a light and waterproof device attached to the chest with two standard electrocardiogram electrodes (Bio Protech Inc, Wonju, South Korea) [2]. The monitor was set to record heart rate and body movements in 60-second epochs. The children were instructed to carry on with their usual behavior and to wear the monitor during all daily activities, including sleep, shower, sauna and swimming. The activity patterns of school-aged children are known to vary markedly between weekdays and weekend days. The children were therefore requested to wear the monitor continuously for a minimum of four consecutive days, including two weekdays and two weekend days, to obtain representative information on physical activity and sedentary time.

Upon retrieving the monitor, the free-living heart rate data were first pre-processed to eliminate potential noise [3], then individually calibrated with parameters obtained from maximal exercise tests performed previously for children participating in this study [4] and finally combined with the body movement sensor data in a branched equation model [5] to calculate instantaneous physical activity energy expenditure (in J × kg^{-1} × min^{-1}) that has been found to agree well with that measured using indirect calorimetry [6, 7]. Physical activity intensity was expressed in standard metabolic equivalents (METs) by defining one MET as an energy expenditure of 71 J × kg^{-1} × min^{-1} or oxygen uptake of 3.5 ml × kg^{-1} × min^{-1}. Data segments with a continuous zero body movement lasting for at least 90 minutes were classified

as ‘non-wear’ if also accompanied by non-physiological heart rate data, i.e. a consistently high Bayesian error [3]. Average total physical activity energy expenditure (in kJ × kg^{-1} day^{-1}) and time spent at multiple intensity levels up to 10 METs was summarized, whilst minimizing potential diurnal bias by wear time imbalance [8]. The method for assessing average total physical activity energy expenditure has been successfully validated against total physical activity energy expenditure during free-living measured by the doubly-labelled water technique in UK men and women with a correlation of 0.67 between these two measures and with no mean bias [9].

Light, moderate and vigorous physical activity were defined as time spent at intensity >1.5 and ≤4.0 METs, >4.0 and ≤7.0 METs and >7.0 METs, respectively. Moderate-to-vigorous physical activity was calculated by summing moderate and vigorous physical activity. Total sedentary time was defined as time spent at intensity ≤1.5 METs excluding sleep. Sleep duration was analyzed from the recordings by a trained exercise specialist and confirmed by a physician, if necessary. The time of falling asleep was defined as movement sensor counts decreasing to zero and heart rate to a plateau low level. The time of waking up was defined as the movement sensor counts increasing and remaining above zero and heart rate increasing and remaining above the plateau level. Data were accepted for the statistical analyses if there was a minimum of 48 hours of activity recording in weekday and weekend day hours that included at least 12 hours from morning (3 am - 9 am), noon (9 am - 3 pm), afternoon (3 pm - 9 pm) and night (9 pm - 3 am) to avoid potential bias from over-representing specific times and activities of the days.

Assessment of dietary factors

Dietary factors reflecting the goals of the intervention, including the consumption of vegetables, fruit and berries, high-fibre (≥5%) grain products, low-fibre (<5%) grain products, high-fat (≥60%) vegetable-oil based spreads, vegetable oils, butter-based spreads, high-fat

(≥1%) milk, low-fat (<1%) milk, red meat, fish and foods with high sugar content, were assessed using a 4-day food record filled out by the parents. The records covered four predefined and consecutive days, including two weekdays and two weekend days or three weekdays and one weekend day [10]. Two food records (0.5%) at 2 year follow-up covered three days and consisted of two weekdays and one weekend day, and their data were also included in the statistical analyses. Clinical nutritionists checked the filled food records together with the family and added any missing information. Food consumption and nutrient intake were assessed using the Micro Nutrica® dietary analysis software, Version 2.5, based on detailed information about the nutrient content of foods in Finland and other countries [11]. Moreover, the clinical nutritionists updated the software by adding new food items and products with their precise nutrient content based on new data in the Finnish food composition database [12] or received from the producers.

We used the Finnish Children Healthy Eating Index (FCHEI), which has been reported to describe well diet quality in children [13], as an indicator of a healthy diet. FCHEI was calculated by summing the reported consumption of five food groups based on their quantiles in the study population [10]. These food groups included vegetables, fruit and berries (scored 1-10); high-fat (≥60%) vegetable oil-based spreads and vegetable oils (scored 0-10); low-fat (<1%) milk (scored 0-9); fish (scored 0-6); and foods with high sugar content (sugar-sweetened beverages, fruit juice, candies, chocolate, added sugar, ice cream, puddings, pastries and biscuits (reverse scored 10-1). FCHEI thus ranged between 2 and 45, a higher score indicating a higher diet quality.

Measurement of body size and composition

Body height and weight were assessed in the morning the children having fasted for 12 hours [1, 10]. Body height was measured three times using a calibrated wall-mounted stadiometer to an accuracy of 0.1 cm the children standing in the Frankfurt plane without shoes. The mean of the nearest two values was used in the analyses. Body weight was measured twice using a weight scale integrated into the Inbody 720® bioelectrical impedance device (Biospace, Seoul, South Korea) to an accuracy of 0.1 kg the children having emptied the bladder and wearing light underwear. The mean of the two values was used in the analyses. Body mass index (BMI) was calculated by dividing body weight (kg) with body height (m) squared. Age- and sex-standardized body height–standard deviation score (SDS) and BMI-SDS were calculated using Finnish references [14]. Overweight and obesity were defined using the International Obesity Task Force criteria corresponding to an adult BMI cut-point at 25 for overweight and at 30 for obesity [15]. Body fat percentage (BF%) and lean body mass were measured using the Lunar® dual-energy X-ray absorptiometry device (Lunar Prodigy Advance; GE Medical Systems, Madison, WI, USA) the children being at a non-fasting state, having emptied the bladder and lying in light clothing with all metal objects removed.

Sample size calculations

Our sample size calculations were based the effects of a dietary intervention on fasting serum insulin and HOMA-IR among children in the Special Turku Coronary Risk Factor Intervention Project (STRIP) [16]. Because of a larger number of children in our study than in the STRIP study, we approximated a slightly smaller difference for the change in fasting serum insulin and HOMA-IR of 0.3 SD between the intervention group (60% of children) and the control group (40% of children) with a power of 80% and a two-tailed p-value for the difference between the groups of 0.05, allowing for a 20% loss to follow-up or missing data. However, these calculations did not allow for non-independence within schools, and therefore the power

could be lower than 80%. According to our calculations, we resulted in a sample size of at least 275 children in the intervention group and at least 183 children in the control group at baseline.

Statistical methods

The formula for the linear mixed-effects model was as follows: $OUTCOME_{it} = (\beta_0+u_i) + \beta_2\ sex + \beta_1\ age + \beta_3\ pubertal\ status + (\beta_4+v_i)\ time + \beta_5\ study\ group\ x\ time + \varepsilon_{it}$, where $OUTCOME_{it}$ are the observations for subject (*i*) at baseline and follow-up (*t*); β_0 is the intercept; u_i is the random subject-specific intercept, β_1, β_2, β_3, β_4 and β_5, are fixed regression coefficients for *sex, age, pubertal status, time and study group x time interaction*, respectively; v_i is the corresponding random slope for follow-up time; and ε_{it} is the error for subject *i* at time *t*. We used the true follow-up time in days as *time* to consider the small variation in the follow-up time that could have caused variation in the changes of the outcome variables during follow-up among the children.

We used the Bayesian information criterion as a measure of model adequacy, a lower value indicating a better model with optimal balance between complexity and good fit. We a priori decided to choose the model with the lowest value of the Bayesian information criterion as the final model for each variable. We fitted all possible models by allowing or ignoring possible clustering on subject or school level for each dependent variable. Thus, we did not force the three-level data structure to our model, because it may not have improved model fit but may have resulted in unnecessary complexity. The data for fasting serum insulin, fasting plasma glucose and HOMA-IR showed the best fit with a model in which the random subject-specific intercept and the random regression coefficient of time were modeled on a subject level by using an independent variance structure, but no random effect for intercept or regression coefficient of time on the school level was included.

References

1. Väistö J, Haapala EA, Viitasalo A, et al. Longitudinal associations of physical activity and sedentary time with cardiometabolic risk factors in children. Scand J Med Sci Sports 2019;29:113-123. doi: 10.1111/sms.13315.
2. Brage S, Brage N, Franks PW et al. Reliability and validity of the combined heart rate and movement sensor Actiheart. Eur J Clin Nutr 2005;59:561-570.
3. Stegle O, Fallert SV, MacKay DJC, Brage S. Gaussian process robust regression for noisy heart rate data. IEEE Trans Biomed Eng 2008;55:2143–2151. doi: 10.1109/TBME.2008.923118.
4. Lintu N, Tompuri T, Viitasalo A, et al. Cardiovascular fitness and hemodynamic responses to maximal cycle ergometer exercise test in children 6-8 years of age - The PANIC Study. J Sports Sci 2014;32:652-659. doi: 10.1080/02640414.2013.845681.
5. Brage S, Brage N, Franks PW, et al. Branched equation modeling of simultaneous accelerometry and heart rate monitoring improves estimate of directly measured physical activity energy expenditure. J Appl Physiol. 2004;96:343–351. doi: 10.1152/japplphysiol.00703.2003.
6. Strath SJ, Brage S, Ekelund U. Integration of physiological and accelerometer data to improve physical activity assessment. Med Sci Sports Exerc 2005;S563–S571. doi: 10.1249/01.mss. 0000185650.68232.3f.
7. Thompson D, Batterham AM, Bock S, et al. Assessment of low-to-moderate intensity physical activity thermogenesis in young adults using synchronized heart rate and Accelerometry with branched-equation modeling. J Nutr. 2006;136:1037–1042. doi: 10.1093/jn/136.4.1037.
8. Brage S, Westgate K, Wijndaele K, et al. Evaluation of a method for minimising diurnal information bias in objective sensor data. In: ICAMPAM (Amherst); 2013.

9. Brage S, Westgate K, Franks PW, et al. Estimation of free-living energy expenditure by heart rate and movement sensing: a doubly-labelled water study. PLoS One. 2015;10:1–19. doi: 10.1371/journal.pone.0137206.
10. Eloranta A-M, Schwab U, Venäläinen T, et al. Dietary quality indices in relation to cardiometabolic risk among Finnish children aged 6–8 years – the PANIC study. Nutr Metab Cardiovasc Dis 2016;26:833-841. doi:10.1016/j.numecd.2016.05.005.
11. Rastas M, Seppänen R, Knuts LR, et al. Nutrient Composition of Foods. The Social Insurance Institution of Finland, 1997.
12. National Institute for Health and Welfare, Nutrition Unit. Fineli. Finnish food composition database. Release 19. Helsinki 2018.
13. Kyttälä P, Erkkola M, Lehtinen-Jacks S, et al. Finnish Children Healthy Eating Index (FCHEI) and its associations with family and child characteristics in pre-school children. Public Health Nutr 2014;17:2519–2527.
14. Saari A, Sankilampi U, Hannila ML, et al. New Finnish growth references for children and adolescents aged 0 to 20 years: Length/height-for-age, weight-for-length/height, and body mass index-for-age. Ann Med 2011;43:235-248.
15. Cole TJ, Bellizzi MC, Flegal KM, et al. Establishing a standard definition for child overweight and obesity worldwide: International survey. BMJ 2000;320:1240-1243.
16. Kaitosaari T, Rönnemaa T, Viikari J, et al. Low-saturated fat dietary counseling starting in infancy improves insulin sensitivity in 9-year-old healthy children: The Special Turku Coronary Risk Factor Intervention Project for Children (STRIP) study. Diabetes Care 2006;29:781-785.

ESM Table 1. Six intervention visits during the 2 year follow-up

Intervention visits	Topics of intervention visits
0.5 months after baseline	Introduction to the contents of the intervention<br>Overview of a physically active lifestyle and a healthy diet
1.5 months after baseline	Supporting total and particularly unsupervised physical activity and diversity of physical activity<br>Supporting the main components of a healthy diet
3 months after baseline	Supporting a healthy lifestyle by utilising information on physical activity, sedentary time, diet, sleep and cardiometabolic risk factors at baseline<br>Increasing total and particularly vigorous physical activity<br>Decreasing sedentary time<br>Improving sleep behavior<br>Increasing the consumption of vegetables and fruit and increasing the intake of dietary fibre
6 months after baseline	Increasing physical activity in everyday life and together with the family<br>Improving overall diet by utilising information on diet received from a 4-day food record at baseline<br>Improving the quality of snacks and decreasing the consumption of sugary foods<br>Improving oral hygiene and health
12 months after baseline	Improving neuromuscular and cardiorespiratory fitness by utilising information on physical activity and cardiorespiratory and neuromuscular fitness at baseline<br>Reducing the intake of saturated fat and increasing the intake of unsaturated fat<br>Increasing the consumption of fish
18 months after baseline	Increasing physical activity and improving cardiorespiratory and neuromuscular fitness to enhance cardiometabolic health by utilising information on cardiometabolic risk factors at baseline<br>Reducing the intake of sodium

ESM Table 2. Goals of the physical activity and dietary intervention

1. Increase total physical activity by emphasizing its diversity
2. Decrease total sedentary time and particularly screen-based sedentary time
3. Decrease the consumption of considerable sources of saturated fat and particularly high-fat dairy and meat products
4. Increase the consumption of considerable sources of unsaturated fat and particularly high-fat vegetable oil-based spreads, vegetable oils and fish
5. Increase the consumption of vegetables, fruits and berries
6. Increase the consumption of considerable sources of fibre and particularly whole grain products
7. Decrease the consumption of considerable sources of sugar and particularly sugar-sweetened beverages, sugar-sweetened dairy products and candy
8. Decrease the consumption of considerable sources of salt and the use of salt in cooking
9. Avoid excessive energy intake

ESM Table 3. Characteristics of children in the intervention and control group at baseline and 2 year follow-up

	Baseline			2 year follow-up		
	Intervention group (*n*=306)	Control group (*n*=198)	*P*-value	Intervention group (*n*=261)	Control group (*n*=176)	*P*-value
Sex			0.520			0.520
Boys, *n* (%)	162 (52.9)	99 (50.0)		162 (52.9)	99 (50.0)	
Girls, *n* (%)	144 (47.1)	99 (50.0)		144 (47.1)	99 (50.0)	
Age, years	7.6±0.4	7.6±0.4	0.989	9.8±0.4	9.8±0.5	0.856
Pubertal status, *n (%)*			0.588			0.968
Tanner stage 1	298 (97.4)	194 (98.0)		192 (76.8)	130 (76.5)	
Tanner stage 2	8 (2.6)	4 (2.0)		58 (23.2)	40 (23.5)	
Body weight, kg	27.0±4.8	26.8±5.3	0.783	34.2±6.8	34.4±8.0	0.897
Body height, cm	128.9±5.5	128.6±5.9	0.847	140.6±5.9	140.2±6.9	0.872
Body height-SDS	0.15±0.99	0.12±1.04	0.713	0.13±0.95	0.06±1.04	0.509
BMI-SDS	-0.16±1.06	-0.20±1.11	0.658	-0.14±1.05	-0.11±1.08	0.797
Body weight status, n (%)			0.596			0.801
Normal weight	264 (86.3)	173 (87.4)		217 (83.1)	144 (81.8)	
Overweight	30 (9.8)	15 (7.6)		37 (14.2)	25 (14.2)	

Obesity	12 (3.9)	10 (5.1)		7 (2.7)	7 (4.0)	
BF%	19.8±8.3	19.9±8.2	0.893	23.3±9.2	23.5±9.3	0.917
Lean body mass, kg	20.7±2.4	20.5±2.5	0.784	24.8±3.1	24.7±3.2	0.971
Total PA energy expenditure, kJ × kg^{-1} day^{-1}	101±32	95±34	0.514	86±29	81±30	0.364
Light PA, h/day	8.6±1.8	8.3±1.8	0.216	6.81.4	6.3±1.5	0.471
Moderate-to-vigorous PA, h/day	2.0±1.0	1.8±1.1	0.648	1.7±0.9	1.6±1.0	0.494
Sedentary time, h/day	3.8±2.0	4.1±2.3	0.362	6.3±1.7	6.5±1.8	0.438
FCHEI	23.6±6.9	22.6±7.0	0.269	25.7±7.3	21.7±6.9	0.002
Food consumption, g/day						
Vegetables, fruit and berries	203±114	219±119	0.159	214±117	190±109	0.055
High-fibre (≥5%) grain products[a]	63±39	62±40	0.909	76±46	73±46	0.474
Low-fibre (<5%) grain products[b]	113±54	115±51	0.644	109±73	104±55	0.576
High-fat (60-80%) vegetable-oil based spreads	6.9±7.6	7.7±8.6	0.362	17.2±14.8	10.3±13.2	0.001
Vegetable oils	4.3±4.4	3.8±3.8	0.271	4.5±4.8	4.3±4.8	0.871
Butter-based spreads	5.8±7.2	6.1±7.2	0.665	4.7±7.5	8.0±9.7	0.003
High-fat (≥1%) milk	170±211	222±243	0.019	96±148	189±231	0.001
Low-fat (<1%) milk	370±289	393±299	0.441	403±296	448±279	0.240
Red meat	56±29	58±34	0.442	60±37	63±36	0.431

Fish	15±20	16±23	0.807	18±23	17±25	0.575
Foods with high sugar content[c]	184±135	207±147	0.275	200±139	219±166	0.478
Total energy intake, mJ/day	6.8±1.3	7.0±1.3	0.254	7.1±1.5	7.0±1.4	0.699
Percentage of daily energy intake						
Carbohydrate intake	52.0±4.8	51.5±5.4	0.847	50.7±5.0	50.0±5.2	0.256
Sucrose intake	12.7±3.6	12.7±3.7	0.932	11.3±3.8	11.8±4.2	0.272
Total fat intake	29.7±4.8	30.4±5.3	0.516	31.0±5.1	32.0±5.2	0.148
Saturated fat intake	12.0±2.7	12.4±2.9	0.520	11.6±2.5	12.6±2.6	0.003
Monounsaturated fat intake	9.9±1.7	10.1±2.0	0.588	10.9±2.1	10.9±2.2	0.921
Polyunsaturated fat intake	4.9±1.2	4.9±1.3	0.734	5.8±1.9	5.6±1.7	0.337
Protein intake	16.9±2.4	16.7±2.5	0.481	16.9±2.7	16.8±2.3	0.501
Fibre intake, g/day	14.5±4.1	14.4±4.0	0.932	15.7±4.7	14.3±4.2	0.010

The values are unadjusted means ± SD for continuous variables and *n* (%) for categorical variables

Baseline data on sex, age, pubertal status, body weight, body height, body height, BMI-SDS and body weight status were available for all 306 children in the intervention group and for all 198 children in the control group. Baseline data on variables were available for the following numbers of children in the intervention group and control group, respectively: BF% and lean body mass 298 and 195; total physical activity energy expenditure 290 and 188; light physical activity and moderate-to-vigorous physical activity 276 and 175; sedentary time 274 and 175; and dietary factors 256 and 167

Two year follow-up data on sex were available for 306 children in the intervention group and for 198 children in the control group. Two year follow-up data on variables were available for the following numbers of children in the intervention group and control group, respectively: age, body weight, body height, body height-SDS, BMI-SDS and body weight status 261 and 176; pubertal status 250 and 170; BF% and lean body mass 248 and 169; total physical activity energy expenditure 224 and 159; light physical activity and moderate-to-vigorous physical activity 218 and 156; sedentary time 216 and 154; dietary factors 231 and 158

The food consumption and total energy intake, macronutrient intake and dietary fibre intake were calculated from reported food consumption from 4 day food records filled out by the parents or caregivers of the children

p values are shown for differences between the intervention and control group from linear mixed-effects models with cluster-robust SEs, except that numbers (percentages) for body weight status and *p* values for their differences between the intervention and control group are from generalised linear mixed-effects models with ordered structure, to account for the clustering effect of schools. Differences with *p* values <0.05 were considered statistically significant

[a] Wholegrain pasta, rice and oatmeal
[b] White pasta, rice and flour
[c] Sugar-sweetened beverages, fruit juice, candies, chocolate, added sugar, ice cream, puddings, pastries and biscuits
